# Medical Department of the Navy

**Published:** 1850-12

**Authors:** 


### MEDICAL DEPARTMENT OF THE NAVY.

A board of surgeons for the examination of assistant surgeons for promotion, and of candidates for admission, will assemble at the Naval Asylum in Philadelphia, on the 16th inst. The following surgeons constitute the board. President, Dr. Dillard. Drs. Horner, Mosely, McLanaghan, and Hunter, members.

